# Management of Fetal Bronchogenic Lung Cysts: A Case Report and Short Review of Literature

**DOI:** 10.1155/2010/751423

**Published:** 2010-03-21

**Authors:** Ülkü Özmen Bayar, Varîm Numanoğlu, Sibel Bektaş, Hakan Sade, Duygu Tatlî

**Affiliations:** ^1^Department of Obstetrics & Gynecology, Karaelmas University Medical School, Kozlu, 67600 Zonguldak, Turkey; ^2^Department of Pediatric Surgery, Karaelmas University Medical School, Kozlu, 67600 Zonguldak, Turkey; ^3^Department of Pathology, Karaelmas University Medical School, Kozlu, 67600 Zonguldak, Turkey

## Abstract

Congenital malformations of the lung (CML) are rare with similar embryological and clinical spectra and could result in mortality if left untreated. Bronchogenic cysts are formed during the budding of the tracheal diverticula and ventral foregut in the embryological period. In this paper we want to present a case of bronchogenic cyst with continuous intrauterine cyst aspiration follow-up. After the baby birth was operated and the postoperative period was uneventful. The pathological examination revealed a bronchogenic cyst.

## 1. Introduction

Congenital lung pathology is uncommon but mostly life-threatening malformations and needs urgent surgery [1, 2], and commonly these malformations share similar embryologic and clinical characteristics [3]. The experience with antenatal detected cystic lung lesions was definitely limited, consisting of case reports [4–6].

It has been supposed that the removal of an intrathoracic mass effect may allow the development of enough lung tissue to maintain postnatal survival. Thoracocentesis, fetal thoracoamniotic shunts, and fetal surgery have been used in human fetuses for this purpose [7–9]. Primary hydrothoraces and congenital pulmonary cystic malformations are agreeable to such treatment. However, these methods carry the potential risks of a procedure-associated morbidity and mortality [10] Presently, there is no combined professional agreement on *inutero* treatment for these conditions.

In this case, intrauterine pulmonary drainage for the management of a congenital bronchogenic pulmonary cyst diagnosed at the 24th gestational week was reported.

## 2. Case Report

**Figure 1:**
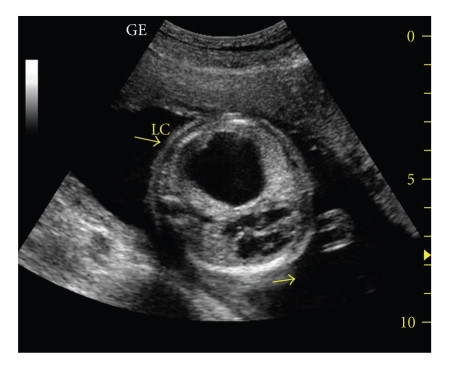
Sonographic examination revealed uniloculated, 40 × 37 mm sized, smooth contour congenital cystic lung lesion which originated from left hemi thorax.

A 25-year-old primigravida woman was referred for further investigation at the 24 weeks' gestation because of congenital cystic lung lesion. A review of the history revealed that the parents were both healthy. The mother did not report any past history of medical problems or recent infectious episodes. In addition, she had no exposure to teratogenic agents such as drugs, tobacco, or alcohol. The family history was also noncontributory. A level II sonographic examination revealed uniloculated, 40 × 37 mm sized, smooth contour congenital cystic lung lesion which originated from left hemi thorax with mediastinal shift causing decrease in lung volume (Figure 1). The other organs were revealed as normal. After discussion with the family about possible outcomes pregnancy was continued. The pediatric surgical team was occupied after the confirmation of an antenatal diagnosed cystic lung disease to assist with antenatal counseling and postnatal management.

**Figure 2:**
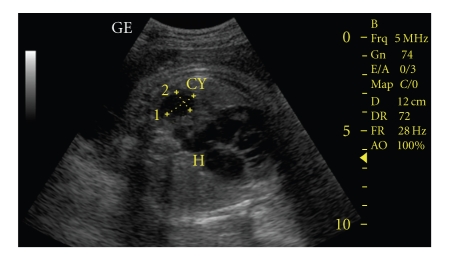
Sonographic examination of congenital cystic lung lesion after thoracocentesis.

**Figure 3:**
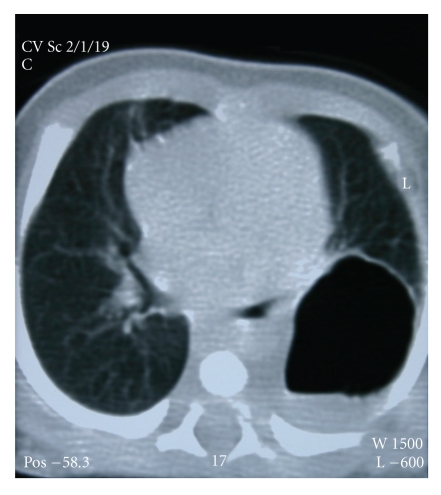
CT imaging of the ballooning of the cyst after birth.

**Figure 4:**
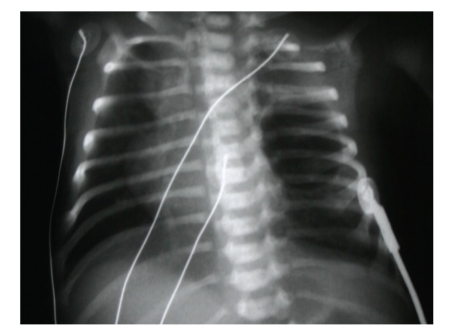
Roentgen imaging of the lungs after birth.

After discussion, *inutero* drainage of the cyst was decided and a written permission form was taken from the family. Pulmonary drainage was made every week from 24 to 30 gestational weeks (Figure 2). We made the procedure with sonographic guidance(LOGIQ 7 scanner GE medical system, Milwaukee, WI). Cyst was aspirated with 20 GA amniocentesis needle (Becton, Dickinson and Company, BD, USA). Genetic and histological analyses were made for cystic fluid. Genetic analysis was normal (46, XX). Histological analysis was reported as erythrocytes and degenerated epithelial cells. At 24th gestational week, cyst/ thorax rate was 3/8, after the 30th gestational week, this rate was decreased to 1/10. Steroid was given for lung maturation. After the 30th gestational week, the diameter of cyst was stabilized at 20 × 17 mm and mediastinal shift disappeared and the patient was follow-up to the birth every two weeks. Hydrops was not observed in *inutero* life. At the 39th gestational week, the female baby was born without any complication. Patient was operated due to overexpansion of the cyst at the postpartum forth day (Figures 3 and 4). After operation, the baby was discharged without any complication. The histological examination of the cyst was reported as bronchogenic cyst (cysts which were laid down with ciliated respiratory epithelium at the inner side and contained focal cartilaginous structures at the fibrous wall). She was 7 months old and healthy at the time of writing.

## 3. Discussion

Recent developments of ultrasonography increase the diagnosis of congenital cystic lung lesions. The progression and natural history of congenital cystic lung lesions is inadequately understood. The spectrum of presentation of cystic lung lesions ranges from fetal hydrops and stillbirth, to neonatal respiratory distress, to asymptomatic lesions. But antenatally diagnosed asymptomatic cystic lung lesions can be present later in life with either respiratory problems or malignancy, or as sudden death [11–13].

These cysts may result from compromised interaction between embryologic mesodermal and ectodermal lung components during development. Pulmonary sequestration, congenital cystic adenomatoid malformation (CCAM), congenital lobar emphysema, and bronchogenic pulmonary cysts are the main four congenital cystic lesions in the lung, but share similar embryologic and clinical characteristics [2].

These pathologies may compromise the prenatal development of functioning parenchymal lung tissue. Consequently, pulmonary hypoplasia may develop and lead to significant perinatal morbidity and mortality at birth. Also, fetal hydrops fetalis and polyhydramnios may develop and the risk of prematurity, furthering the risk of perinatal loss, increases.

Bronchogenic pulmonary cysts were one of the most common lesion, and, commonly occur early in lung development [3]. Bronchogenic cysts might accurately be called lung bud cysts, since they possibly originated from embryonic bud tissue before the bronchi appeared. The cyst is mostly laid down with ciliated columnar epithelium and contain cartilaginous, smooth muscle tissue, glandular structures, and sometimes mucosa of esophagus and stomach [3]. Bronchogenic cysts need urgent surgery at a rate of 33.3% [14]. Asymptomatic bronchogenic cysts,operated nearly 5-7 years old are commonly complicated with repeated infection and cough but none shows respiratory distress. 

Percutaneous, *inutero* pulmonary drainage in fetuses with ultrasonographic evidence of congenital pulmonary cystic malformations is associated with improved perinatal survival [10]. *Inutero* pulmonary drainage has been used for many years but no combined treatment consensus has been accomplished. Elective excisional surgery recommended for those infants and children with symptoms, evidence of mediastinal shift, diagnostic doubt, and involvement of a significant part of the lung (>25% of ipsilateral lung, as assessed on CT scan) [12]. Lung preserving surgery is promising only if undertaken electively before any infective complication in antenatally diagnosed thoracic lesions [13].

At postnatal period, as a conclusion prenatal recognition of these relatively rare congenital cystic lung lesions would lead to the immediate, proper surgical intervention and the need for surgery should be based on appropriate postnatal investigations, rather than antenatal behavior. This approach should retrieve almost all the affected cases, which would otherwise face a rather dismal future.
